# Three-dimensional matrix fiber alignment modulates cell migration and MT1-MMP utility by spatially and temporally directing protrusions

**DOI:** 10.1038/srep14580

**Published:** 2015-10-01

**Authors:** Stephanie I. Fraley, Pei-hsun Wu, Lijuan He, Yunfeng Feng, Ranjini Krisnamurthy, Gregory D. Longmore, Denis Wirtz

**Affiliations:** 1 Department of Bioengineering, University of California San Diego, La Jolla, California 92093, USA; 2 Department of Chemical and Biomolecular Engineering, The Johns Hopkins University, Baltimore, Maryland 21218, USA; 3 Johns Hopkins Physical Sciences—Oncology Center, The Johns Hopkins University, Baltimore, Maryland 21218, USA; 4 Departments of Medicine and Cell Biology and Physiology and BRIGHT Institute, Washington University School of Medicine, St. Louis, Missouri 63110, USA; 5 Department of Pathology, Geisel School of Medicine at Dartmouth, Lebanon, New Hampshire 03756, USA

## Abstract

Multiple attributes of the three-dimensional (3D) extracellular matrix (ECM) have been independently implicated as regulators of cell motility, including pore size, crosslink density, structural organization, and stiffness. However, these parameters cannot be independently varied within a complex 3D ECM protein network. We present an integrated, quantitative study of these parameters across a broad range of complex matrix configurations using self-assembling 3D collagen and show how each parameter relates to the others and to cell motility. Increasing collagen density resulted in a decrease and then an increase in both pore size and fiber alignment, which both correlated significantly with cell motility but not bulk matrix stiffness within the range tested. However, using the crosslinking enzyme Transglutaminase II to alter microstructure independently of density revealed that motility is most significantly predicted by fiber alignment. Cellular protrusion rate, protrusion orientation, speed of migration, and invasion distance showed coupled biphasic responses to increasing collagen density not predicted by 2D models or by stiffness, but instead by fiber alignment. The requirement of matrix metalloproteinase (MMP) activity was also observed to depend on microstructure, and a threshold of MMP utility was identified. Our results suggest that fiber topography guides protrusions and thereby MMP activity and motility.

Cell motility through 3D extracellular matrix (ECM) is a key biological process involved in normal development and homeostasis, as well as the progression of diseases such as metastatic cancer. Efforts to understand the invasion and movement of cancer cells through the collagenous ECM surrounding tumors, a key step in metastatic progression, originally used 2D model systems that allow the complexities of the microenvironment to be significantly decomposed. Now, however, multiple studies have highlighted the major differences between 2D and 3D cancer cell motility^1^,^2^,^3^,^4^, and 3D systems have become standard. Within these 3D systems, several physical features of the extracellular matrix (ECM), i.e. stiffness^5^,^6^,^7^,^8^, ligand density^9^,^10^,^11^, crosslinking^6^, and pore size^12^,^13^, and fiber alignment^14^,^15^,^16^ have been implicated independently as driving factors of cancer cell motility. Most studies have focused on only one or two matrix parameters, despite the fact that a change to any one parameter almost always affects another, or they have used non-native polymers or digested ECM proteins that do not crosslink and form microstructures that are physiologically relevant^17^. An integrated understanding of how density (which is the most commonly used descriptor), ligand presentation, crosslinking, and microstructural organization are related to each other and to cell behavior is still lacking in the context of the native acid extracted collagen-based 3D ECM now used by many researchers.

Here we take an integrative approach to characterizing and understanding these convolved features by embracing the complex combinations of matrix parameters that arise naturally in 3D self-assembling collagen I networks. By creating collagen gels of increasing density over a six-fold rage, we generated multiple complex matrix features. Embedded cells were assessed for their motility behavior (cell speed, invasion distance, and protrusion dynamics) while the matrix itself was characterized for its physical features (stiffness, density, pore size, and alignment of fibers). Then additional enzymatic crosslinking achieved changes in matrix parameters independently of density changes. Cross correlations among these measurements allowed us to uncover a distinct relationship between fiber alignment and cell motility independent of pore size and bulk matrix stiffness. Central to our approach is the fact that in 3D collagen, cancer cells move into the 3D matrix, rarely retracing the void tracks they leave behind, and so are always interacting with consistent microstructural properties^1^,^18^ (see also Results section).

## Results

### 3D cell motility is biphasic with increasing collagen density

**Figure 1:**
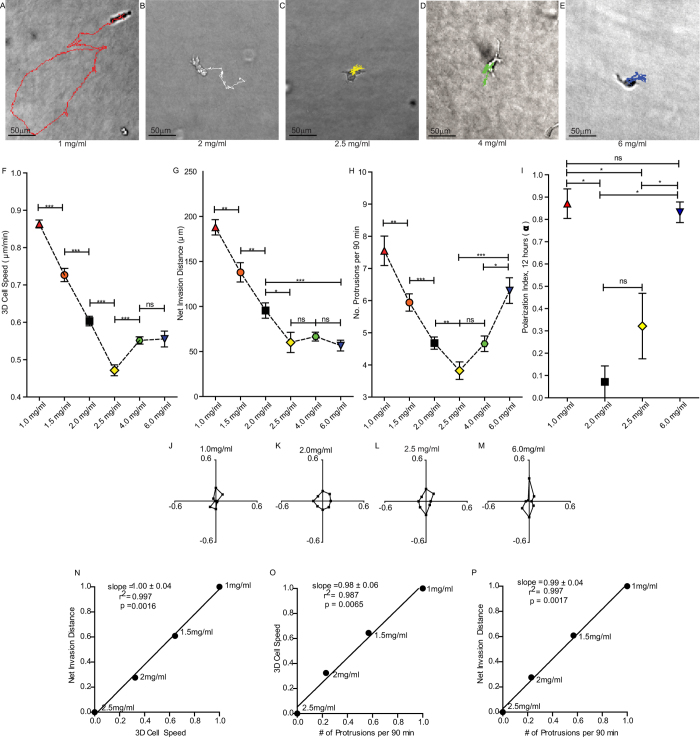
Cell motility parameters display a bi-phasic trend as collagen density is increased in a 3D matrix. (**A–E**) Micrographs of typical single cells in varying concentrations of collagen matrix with 16.5 h cell migration trajectories overlaid. (**F**) Average speed of cells embedded in 3D matrices of increasing collagen density. (**G**) Average net invasion distance of cells migrating through varying concentrations of 3D collagen matrices over 16.5 h. (**H**) Average number of protrusions per 90 min generated by cells migrating through matrices of increasing collagen density. (**I**) Average polarization index for seven randomly chosen cells in each matrix condition over 12 h; a value of one indicates that cells remain polarized in their initial orientation and a value of zero indicates that cells repolarize equally in all directions. (**J–M**) Orientation of protrusions plots showing fraction of cellular protrusions extended in each direction during migration in 1, 2, 2.5 and 6 mg/ml collagen matrices. Fractions calculated from average of seven randomly chosen cells monitored for 12 h each. (**N**–**P**) Correlation plots of min-max normalized invasion distance vs. 3D cell speed, 3D cell speed vs. number of protrusions per 90 min, and invasion distance vs. number of protrusions per 90 min. N = 3 independent repeats for each graph; at least 20 cells were analyzed per repeat. Correlation plots show slope, square of Pearson’s correlation coefficient, and p value of the linear dependence between the two normalized axis variables. Error bars represent s.e.m. ***p < 0.001; **p < 0.01; *p < 0.05

We first asked what differences in cell motility where characteristic of increases in ligand density in 3D collagen. Cell motility parameters, including speed, invasion distance, and number and orientation of protrusions of embedded HT-1080 human fibrosarcoma cells were systematically assessed as collagen I density was increased from 1-6 mg/ml. Interestingly, a biphasic dependence of multiple motility parameters with collagen I density was observed, which is opposite to what occurs for 2D cell motility with increasing ligand density^9^,^19^ and for what has been predicted for 3D matrices^20^. At low collagen I concentration in 3D (1 mg/ml), cells moved rapidly and persistently with a sustained high rate of protrusion formation, and invaded to distances far from their point of origin (Fig. 1A,F–H). Cells also maintained the orientation of their protrusions over 12 h (Fig. 1I,J), i.e. the large majority of cell protrusions remained polarized along the original axis of elongation of the cell. At intermediate collagen concentrations (2 and 2.5 mg/ml), in contrast to what would have been predicted from cells moving on a 2D substrate, cells migrated more slowly and invaded smaller distances from their point of origin than cells in 1 mg/ml matrices (Fig. 1 B,C,F,G). Cells also generated fewer protrusions (Fig. 1H) and the directionality of their protrusions was significantly more isotropic than cells in 1 mg/ml matrices (Fig. 1I,K,L). Finally, when cells were embedded in high-density collagen matrices (4 and 6 mg/ml), cell speed increased, but did not achieve speeds observed in 1 mg/ml matrices (Fig. 1D–G). Cells also increased their rate of protrusion formation (Fig. 1H) and became highly polarized once again (Fig. 1I,M), similarly to cells in 1 mg/ml matrices. MDA-MB-231 human breast cancer cells were used to test whether this motility response to increasing 3D collagen density was cell-type specific. Highly similar motility trends were observed for MDA-MB-231 cells (Supplementary Fig. 1A).

Cell migration speed, invasion distance, and the number of cellular protrusions were highly correlated with each other and with collagen density from 1–2.5 mg/ml (Fig. 1N–P). However, data for the higher concentrations of 4 and 6 mg/ml shifted away from this trend and were considered outliers (Supplementary Fig. 1D–F). To determine if properties of the matrix other than collagen density were involved in modulating cell motility and to understand the switch in behavior that occurred for 4–6 mg/ml densities, we next characterized the physical and architectural properties of each matrix.

### Fiber alignment and pore size, but not matrix stiffness, correlate with cell motility

**Figure 2:**
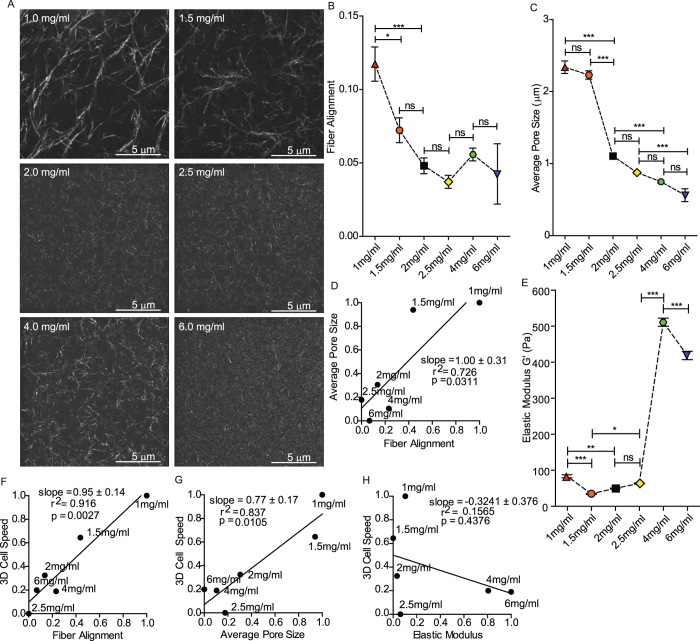
Influence of matrix microstructure on cell motility. (**A**) Reflection confocal micrographs of 3D collagen matrices of increasing collagen density, scale bar is 5 μm. (**B**) Average extent of collagen fiber alignment measured by FFT analysis in matrices of varying concentrations of collagen. (**C**) Average pore size of collagen fibers in matrices of varying density. (**D**) Correlation plot of normalized average pore size vs. alignment of fibers. (**E**) Global shear elastic modulus of collagen matrices of varying densities. (**F**) Correlation plot of normalized 3D cell speed vs. alignment of fibers. (**G**) Correlation plot of normalized 3D cell speed vs. average pore size. (**H**) Correlation plot of normalized 3D cell speed vs. shear elastic modulus. N = 3 independent gelation repeats for each graph of matrix characteristics; images of at least 5 different positions within the central region of the matrix, far from the container walls, were used from each repeat. Error bars represent s.e.m. ***p < 0.001; **p < 0.01; *p < 0.05.

Since several physical properties of the matrix (pore size, stiffness, fibrilar structure, etc.) can change concurrently with changes in collagen density, we aimed to characterize these finer physical details and ask how these features varied with each other and with observed motility responses. Analysis of reflection confocal images of 1–6 mg/ml matrices (Fig. 2A) showed that fiber alignment (measured at length scales of the cell) varied biphasically with collagen concentration (Fig. 2B). The average pore size varied somewhat irregularly, but overall was reduced with increasing collagen concentration (Fig. 2C). These two matrix parameters correlated significantly with one another across all collagen densities (Fig. 2D). Quantitative shear rheometry was used to measure the bulk matrix elasticity, which varied somewhat irregularly with increasing collagen concentration (Fig. 2E). Although previous studies suggest that the elastic modulus should scale positively with increasing collagen concentration^21^,^22^, it is critical to note that the process by which collagen is extracted^23^, the gelation conditions^22^,^24^, and even the thickness of the matrix^22^ can all significantly alter its mechanical properties and related microstructure^25^. Collagen density alone, which is often the only information provided in studies, is not sufficiently descriptive to enable direct comparisons. In our collagen matrices, the alignment of fibers decreased significantly when collagen density was increased from 1 mg/ml to 1.5 mg/ml, but the average pore size did not change. That the mesh is not tighter, but is significantly less aligned likely explains the decrease in the elastic modulus from 1–1.5 mg/ml. Then from 1.5 to 2 mg/ml pore size decreases dramatically while fiber alignment drops only slightly. This means that 2 mg/ml represents a tighter mesh, which would be expected to increase the elastic modulus compared to 1.5 mg/ml. Comparing the measured changes in matrix microstructure and mechanics to changes in cell motility revealed a significant correlation of cell speed and invasion distance with both fiber alignment and pore size (Fig. 2F,G) across the varying densities of collagen. Cell speed and invasiveness did not correlate with matrix stiffness, at least within the range of collagen concentrations tested (Fig. 2H).

### Additional crosslinking reinforces fiber alignment as a predictive parameter

**Figure 3:**
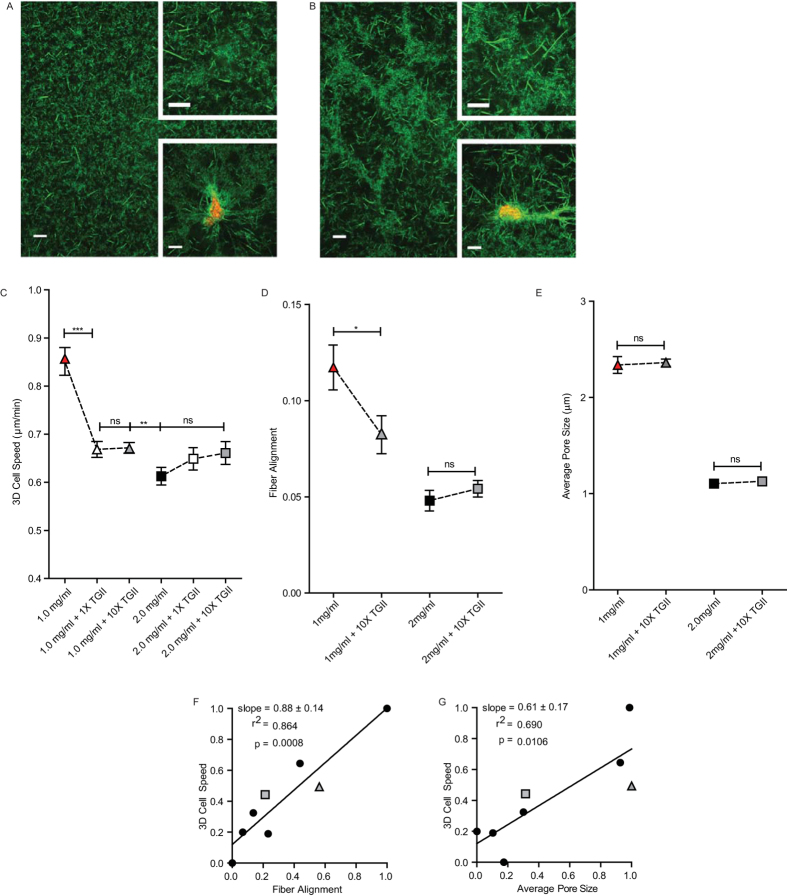
Additional crosslinking with TGII alters matrix microstructure and cell motility but leaves global collagen density unchanged. (**A**,**B**) Reflection confocal micrographs of 2 mg/ml collagen matrix and 2 mg/ml collagen matrix further crosslinked with TGII, respectively, at three magnifications (background image, top inset, and bottom inset). Bottom insets show embedded cells labelled with red membrane marker. (**C**) Average speed of cells migrating through 1 mg/ml collagen or 2 mg/ml collagen with and without the addition of 1X or 10X TGII crosslinking enzyme. (**D**) Average extent of collagen fiber alignment measured by FFT analysis of 1 and 2 mg/ml collagen matrices with and without additional crosslinking by 10X TGII. (**E**) Average pore size of 1 and 2 mg/ml collagen matrices with and without additional crosslinking by 10X TGII. (**F**,**G**) Correlation plots of 3D cell speed vs. alignment of fibers and pore size (from Fig. 2) with 1 mg/ml and 2 mg/ml + 10X TGII data points added (grey triangle and square, respectively). N = 3 independent repeats for (**C**–**E**); at least 20 cells were analyzed per repeat for (**C**) Scale bars in panels (**A**,**B**) are 100 μm, 5 μm, and 10 μm for background image, top inset, and bottom inset respectively. Error bars represent s.e.m. across all data points in all repeats for each condition. ***p < 0.001; **p < 0.01; *p < 0.05.

Since local fiber alignment and pore size correlated with each other across a wide range of collagen densities, we developed a method to alter microstructure independently of density to try to decouple these features. This was accomplished by the addition of the crosslinking enzyme Transglutaminase II (TGII) to the matrices. Crosslinking of 1 mg/ml and 2 mg/ml collagen matrices by the addition of TGII allowed alteration of the microstructural properties of the matrices, while preserving global collagen concentration (Fig. 3A,B). Cells embedded in 1 mg/ml matrices further crosslinked with TGII migrated significantly more slowly than cells in non-TGII treated 1 mg/ml matrices (Fig. 3C). However, the migration of cells embedded in 2 mg/ml matrices further crosslinked with TGII was unchanged compared to non-TGII treated 2 mg/ml matrices (Fig. 3C). Addition of a ten times higher concentration of TGII did not further alter the ECM microstructure or cell speed at either concentration of collagen (Fig. 3C).

As a control to ensure that TGII did not affect cell physiology directly, we applied TGII to cells migrating on conventional 2D substrates at the same concentration as used to crosslink 3D matrices and monitored possible changes in cell behavior. Cell speed on conventional 2D substrates was not altered by the addition of TGII (Supplementary Fig. 1C), suggesting that our observed changes in 3D cell motility were specifically related to crosslinking of the collagen matrix and the ensuing alterations to its physical properties.

Our microstructural analysis of the matrices showed that collagen crosslinking by TGII reduced the alignment of the fibers in 1 mg/ml matrices (Supplementary Fig. 1B), but did not significantly alter the alignment of 2 mg/ml matrices (Fig. 3A,B(top inset)). These results fit the previously established correlation between cell speed, invasion distance, and fiber alignment (Fig. 3F) and further increased the significance of the correlation. However, average pore size was unchanged following the addition of crosslinking TGII for both 1 mg/ml and 2 mg/ml matrices (Fig. 3E,G), indicating that fiber alignment can regulate cell motility independently of pore size.

### Requirement for MMPs critically depends on matrix microstructure

MMPs are thought to be critical for cancer cell migration and invasion in crosslinked matrices where the average pore size is significantly smaller than the cell body. Here cell size is an order of magnitude larger than the average pore size of the matrix, ~0.6–2.3 μm in diameter (Fig. 2C), so we wondered how the requirement for MMPs might be affected. MMP −1, −2, −7, −9, and the membrane tethered MT1-MMP, which are considered critical for cell migration and invasion^26^, were inhibited for cells embedded in either 1 mg/ml or 2.5 mg/ml collagen matrices using a range of Marimastat concentrations. The concentrations of 1 mg/ml and 2.5 mg/ml were selected for comparison because they represent the extremes of both cell motility and fiber alignment observed in this study (Figs 1 and 2). For 1 mg/ml, collagen I formed structures that were the most aligned and for which cells were the most invasive, protrusive, and uni-axially oriented (Fig. 1G,I,J and Fig. 2B). For 2.5 mg/ml, collagen I formed structures that were the least aligned, and for which cells were the least invasive and protrusive, and protrusions were more isotropically oriented (Fig. 1C,G–I,L and Fig. 2B).

**Figure 4:**
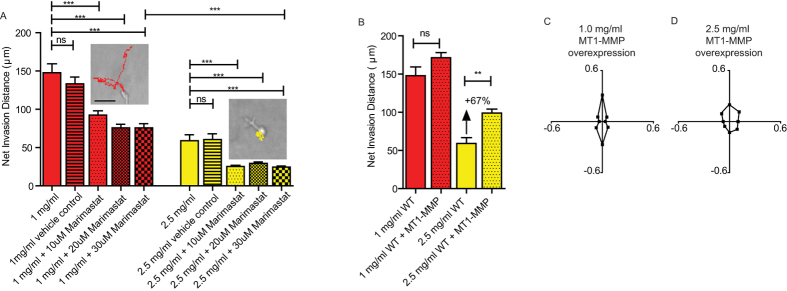
Effect of MMP inhibition on cell invasion depends on matrix conditions. (**A**) Net invasion distance achieved by 1 or 2.5 mg/ml matrix embedded HT-1080 cells treated with vehicle (DMSO), 10 μM, 20 μM, or 30 μM Marimastat MMP inhibitor. Vehicle control concentration is equivalent to the 30 μM drug condition. Inset shows representative cells and their 16.5 h migration trajectories in 1 mg/ml + 30 μM Marimastat (left, red) and 2.5 mg/ml + 30 μM Marimastat (right, yellow). Scale bar is 50 μm. (**B**) Net invasion distance of 1 or 2.5 mg/ml matrix embedded HT-1080 cells with overexpression of MT1-MMP. (**C**) Protrusion orientation of MT1-MMP overexpressing cells in 1 mg/ml matrix over 12 h. (**D**) Protrusion orientation of MT1- MMP overexpressing cells in 2.5 mg/ml matrix over 12 h. N = 3 independent repeats for each condition, at least 20 cells were analyzed per repeat per condition. At least 300 protrusions total across at least 7 cells over 12 h increments were analyzed for each orientation graph. Error bars represent s.e.m. across all data points for each condition. ***p < 0.001, **p < 0.01.

Maximal inhibition with Marimastat at a concentration of 30 μM (higher concentrations resulted in cell death) did not abolish motility for cells in highly aligned 1-mg/ml matrices. Instead, they maintained ~50% of their invasive ability, on average 75μm over 16.5 h (Fig. 4A). Marimastat-treated cells in 2.5-mg/ml matrices (Fig. 4A, right inset) were essentially confined to the void in which they had been initially embedded. When MT1-MMP was overexpressed in cells embedded in 1-mg/ml and 2.5-mg/ml matrices, cell motility increased by an insignificant amount and 67%, respectively (Fig. 4B).

Taken together, these results suggest a threshold for MMP utility within a 1-mg/ml matrix for which collagen fibers form highly aligned microstructures. MT1-MMP overexpression did not alter the orientation of cellular protrusions in these matrix conditions (Fig. 4C, D). This suggests that regardless of whether more MT1-MMP mediated collagen degradation occurs, the directionality of motility remains unaltered and is driven and oriented by the fibrilar topography of the ECM.

## Discussion

We have conducted a multi-parametric quantitative analysis of cell motility in 3D collagen matrices of controlled density to understand how fibrilar matrix properties modulate motility. This study was designed to span a broad range and complexity of physical features of the matrix, including pore size, fiber alignment, stiffness, and extent of crosslinking, such that any key feature would be tested in combination with several features to avoid bias. After a comprehensive quantification of each parameter, we relied on cross-comparison of matrix parameters and motility measurements to identify the features of the matrix influencing cell motility. We find that the local alignment of collagen fibers is highly predictive of cell motility across the entire range and combinations of matrix conditions tested, while collagen density is highly predictive only at low concentrations. Pore size correlates with cell motility parameters, corroborating recently published findings^13^, but less significantly than fiber alignment. By incorporating a secondary method of crosslinking that modulated matrix architecture independently of density, the relationship between fiber alignment and motility was further reinforced while that of pore size, density, and motility was not.

Our collagen image analysis method provides a score for the pore size between fibers and the fiber alignment for fiber intensity signals that are greater than imaging background noise, which is removed during reflection confocal image processing (see Methods). Moreover, imaging resolution must also be able to resolve fiber locations spatially. In general, reflection imaging is limited in its ability to detect fibers that are oriented at an angle greater than ~50 degrees to the 2D imaging plane^27^ and by the spatial resolution of the imaging system (~0.20 μm/px in our case). If the average space between fibers is less than the spatial resolution of the imaging system, the analysis results can be unreliable. In Fig. 2A, fibers are distinguishable in all conditions, but the 6 mg/ml case shows significantly fewer distinguishable fibers. Additionally, the average pore size in 6 mg/ml approaches the imaging resolution limit (Fig. 2C), indicating a rationale for the larger variability in alignment measurement for the 6 mg/ml case (Fig. 2B) compared to all other conditions. Nonetheless, our method is able to make highly reproducible, quantitative comparisons among the other multiple collagen concentration and crosslinking conditions and to predict the effect of ECM architectural changes on cell motility with high statistical significance (Fig. 3F).

Our alignment analysis constitutes a bulk measurement for all the fibers in the imaging area (analyzed images of collagen cover 61 μm × 61 μm) and reports the degree of anisotropy of the image as a whole. Simulations show that our method for quantifying alignment is not sensitive to changes in the length of fibers, but is sensitive to significant changes in width (data not shown). However, the width of the fibers did not change significantly across the different concentrations of collagen.

The significant influence of fiber alignment, measured at a length scale that is of the same order of magnitude as cell invasion distance in our study, likely arises because of topographic guidance as the cell protrudes and attaches to the ECM. Indeed, we find that the orientation of cellular protrusions follows a similar trend to that of fiber alignment. During review of this manuscript, another paper appeared showing that aligned 3D collagen matrices enhance migrational persistence and orient cellular protrusions, corroborating our findings^28^. Other studies using synthetic nanofiber scaffolds have demonstrated that fiber alignment promotes cell elongation, restricts sites of cell attachment, aligns adhesions, and promotes faster migration speeds^29^. This imposition on cell shape has been shown to play an important role in traction generation on 2D substrates^30^, resisting repolarization on 1D substrates^31^, and in generating polarized traction forces in 3D, which are thought to facilitate invasion^32^. Using a native 3D collagen matrix, our studies identify fiber alignment as a key feature even in light of the introduction of many additional complex matrix changes.

In our study, matrix architecture is assessed without cells present in the matrix. Since cells continuously migrate into the matrix and do not typically turn to retrace the void space they leave behind (over our observation time of 16 h), and because we seed cells at a very low density, we anticipated that cells would continuously encounter the same conditions as dictated by the initial matrix architecture. That these initial matrix conditions predict cell motility indicates that this assumption is justified. The ability of cells to pull on and align the matrix after their initial adhesion to the ECM may also play a role, possibly feeding back to positively reinforce cell polarization. However, others have shown that cell invasion in 3D matrices correlates more strongly with cell shape elongation than with contractility^32^.

It is well established that increasing ligand density on planar 2D substrates induces a biphasic increase and then decrease in cell speed^9^. In 3D matrices, we observe the opposite: a decrease then an increase in cell speed. The 2D phenomenon is thought to reflect changes in adhesion strength mediated by the aggregation of proteins at focal-adhesion sites^33^. At high planar ligand density, strong adhesion inhibits cell movement, while at low planar ligand density, weak adhesion strength causes less efficient motility. Here, the fibrilar topology of the 3D matrices presents a distinct network of nanoscale, linearly organized ligands to which the cell adheres. A recent modeling study addressed this point and predicted differences in cell speed and invasion distance based solely on fibrilar matrix organization, finding a significant role for alignment^34^. Our study shows experimentally that the alignment of discrete ligand-containing fibers directs cell protrusions and motility distinctly in 3D^35^,^36^,^37^.

By treating cells in 3D matrices with Marimastat, a wide-spectrum MMP inhibitor, our studies show a near complete loss of motility for cells in matrices of intermediate density (2.5 mg/ml), where pore size averages 0.9 μm in diameter and where fibers and cell protrusions are isotropic. However, treating cells in low-density matrices (1 mg/ml), where average pore size is still smaller than the cell (2.3 μm) but fibers and protrusions are highly aligned, results in only a 50% reduction in cell invasion distance, indicating MMP independent motility and significant cell deformation occur. Yet, cells maintain a higher level of motility than expected based on previously reported steric limitations of pore size^13^. This could be due to differences in pore size measurement techniques or may indicate that fiber alignment influences cell deformability.

Overexpression of MT1-MMP in our study suggests that a threshold may exist for MMP utility, even in matrices of relatively small pore size. For cells embedded in low-density 1 mg/ml matrices with pores averaging 2.3 μm in diameter, cells move rapidly and invade further than in any other condition. In this case, overexpression of MT1-MMP did not result in any significant increase of invasive ability. Low ECM densities are known to reduce baseline MMP production while high ECM densities feedback to increase baseline MMP production^38^,^39^,^40^,^41^,^42^. This suggests that cells in low-density 1 mg/ml matrices could benefit from overexpression of MT1-MMP, but in fact do not. It is possible that overexpressed MT1-MMP in cells in 1 mg/ml remains inactive. Alternatively, because MMPs are most active in protrusions^43^,^44^, and protrusion number and spatial orientation about the cell body were modulated by matrix architecture, it is possible that aligned fibers optimize the efficient use of MMPs by spatially and temporally focusing the activity over longer time scales. Or aligned fibers may present cleavage sites in a distinct and more susceptible way. Future work will aim to further delineate these mechanisms. It is interesting to note that in our previous work, we discovered that matrix-embedded cells lacking certain focal adhesion proteins display variations in cell protrusion rate, orientation, cell speed, and invasion, which resemble some of the same motility outcomes that were achieved here by simple variations in 3D ECM microstructure. This highlights the fact that physical changes in the ECM can affect the outcomes of cell motility as drastically as genetic changes in cells, and demonstrates the importance of characterizing and further understanding the role of the ECM in modulating cell motility.

## Conclusions

This study shows that the ECM microstructural parameter of fiber alignment reliably predicts multiple features of cancer cell motility in 3D matrices. Fiber alignment modulates cellular protrusion rate and orientation, which regulate cell motility. Fiber alignment varies significantly with collagen density and the extent of matrix crosslinking, highlighting the need for standardizing ECM microstructure characterization, which may help unify outcomes across 3D motility studies. The techniques and results presented here lend further insight into the role of the physical ECM and crosslinking enzymes in cancer cell motility and metastasis.^45^

## Materials and Methods

### Cell Culture

HT-1080 cells (ATCC, Manassas, VA) were cultured in Dulbecco’s Modified Eagle’s Medium (Mediatech Inc., Manassas, VA) supplemented with 10% (v/v) fetal bovine serum (Hyclone Standard, Logan, Utah) and 0.001% (v/v) Gentimicin (Sigma, St. Louis, MO). Cells were passaged every 2–3 days for a maximum of 20 passages. MDA-MB-231 cells (Physical Sciences Oncology Center, NIH) were cultured in Dulbecco’s Modified Eagle’s Medium (Mediatech Inc., Manassas, VA) supplemented with 10% (v/v) fetal bovine serum (Hyclone Standard, Logan, Utah) and 0.1% (v/v) penicillin/streptomycin (Sigma, St. Louis, MO). Cells were passaged every 2–3 days. All cells were maintained at 37 ^o^C and 5% CO_2_ in a humidified environment during culture and imaging.

### MT1-MMP overexpression

Full-length human MT1-MMP (hMT1-MMP) was obtained by overlapping PCR and subcloned into the EcoRI/AgeI sites of pFLRcmv-Ypet-puro vector^46^. The vector was sequence validated prior to lentivirus formation and transduction. Localization and fluorescence indicate expression. Only cells fluorescent with MT1-MMP-Ypet were tracked.

### 3D collagen I gelation, crosslinking, and drug inhibitors

Cell-impregnated 3D collagen matrices were prepared similarly to that described previously^1^,^4^,^47^. Briefly, 20,000 cells suspended in a 1:1 (v/v) solution of culture medium and 10x reconstitution buffer were gently mixed by pipetting with the appropriate amount of low concentration rat tail type I collagen in acetic acid (BD Biosciences) on ice to achieve a 500 μl solution with a final concentration of 1.0, 1.5, or 2.0 mg ml^−1^ collagen. High concentration rat-tail type I collagen in acetic acid (BD Biosciences) was used for 2.5, 4.0, and 6.0 mg ml^−1^ conditions. Then, NaOH (1N) was added in the amount of 3.6% (v/v) of the volume of collagen to initiate polymerization. The mixture was again gently but thoroughly pipetted on ice. At this point, 0.1 μg (1X, 1 μl) or 1 μg (10X, 10 μl) of purified recombinant human Transglutaminase II (TGII) in solution (R&D Systems, Minneapolis, MN) was mixed into the solution for those matrices that were to be additionally crosslinked. Initial medium and reconstitution buffer volumes were adjusted equally to account for the additional volume of TGII. The solutions were mixed again thoroughly on ice, with care taken to avoid introduction of bubbles, and subsequently placed in a standard glass bottom 24-well plate. Immediately, the well plate was placed in a 37 ^o^C incubator for at least 30 min. Additional warm cell culture medium, 500 μl, was then added on top of the matrices and the plate put back into a CO_2_ incubator for full pH buffering. Reproducibility of gel structures relied on consistently using the same dish size (24-well glass bottom plates) plus accurate volumes (good pipetting technique) to ensure reproducible gel thickness and pH, and consistent timing (mixing all ingredients together on ice in less than 30 sec) plus temperature control (keeping all ingredients on ice throughout the process, mixing everything on ice, and then immediately transferring to a temperature and CO_2_ controlled incubator for at least 1 hr gelation time). Since each of the above-mentioned variables can influence gel structure and because time is limited during the gelation procedure, quantification of matrix structure after the full process, using our image analysis methodology presented herein, served as our quality control checkpoint. Statistical analysis of data produced from the image analysis of matrix structure and from cell motility analysis demonstrates the reproducibility of our procedure and the utility of our image analysis method.

For MMP inhibition experiments, Marimastat (AnaSpec Inc., Fremont, CA) or vehicle control DMSO (Santa Cruz Biotechnology Inc., Santa Cruz, CA) was mixed into the medium before it was added on top of the cell impregnated matrix to achieve a final concentration of 10, 20, or 30 μM in the well. Since DMSO itself was found to dramatically effect cell motility at low concentrations (data not shown), we note that making the final amount of DMSO in our experiments optimally minimal required the purchase of dry Marimastat to make high concentration stock solutions. The final concentration of DMSO which did not impair motility, as demonstrated by the vehicle control data, was 0.1% (v/v) DMSO in culture media. This value was used to back-calculate the appropriate mixture of Marimastat to DMSO to achieve the desired stock concentration of inhibitor. Low cell density in the matrices helped to ensure that cells moved as singlets and that motility measurements were accurate.

### Immunofluorescence microscopy and reflection confocal microscopy

To visualize collagen fibers within a 3D collagen gel, both immunofluorescence and reflection confocal images were collected using a Nikon A1 confocal microscope equipped with a 60x water-immersion objective (Nikon) and controlled by Nikon Elements imaging software (NIS-3.1). Collagen matrices imaged with immunofluorescence were first gently detached from the well plate walls, then incubated with primary and secondary antibodies in PBS with 1% BSA for 24 h (Abcam ab24133 anti-collagen I and AlexaFluor secondary). For reflection imaging, the microscope was configured to capture 488 nm light reflected during illumination with the 488 nm laser. Reflection and immunofluorescence images of detached gels were used to validate the fiber analysis algorithm, but only reflection images of attached gels were used for correlation plots.

### Cell tracking and motility

Cells embedded in 3D collagen matrices, far from the walls and bottom of the 24-well plate, were imaged at 10x magnification every 2 min for 16.5 h similar to that which was previously described^1^,^4^. Briefly, cells which were actively moving as singlets over the course of the timelapse were tracked using Metamorph software with the Metavue Track Objects Application (Molecular Devices, Sunnyvale, CA) with x and y position coordinates measured for each 2 min time point, the collection of points comprising the cell’s trajectory. Cell speed was calculated by averaging the distance per 2 min time interval that each cell traveled. Net invasion distance is a measure of the maximum displacement of the cell from its point of origin. This was found by calculating the displacement of each x, y tracking point over 16.5 h from the cell’s original x, y location at 0 h, then finding the maximum of these displacements for each cell. For correlation plots, min-max normalization was used to re-scale the average of each data set between 0 and 1, avoiding units bias in cross-comparisons.

### Cell protrusion analysis

Orientation of cellular protrusions and number of cellular protrusions were calculated as described in detail previously^1^,^4^. Briefly, the position of protrusions at least 5 μm in length from the cell periphery were tracked and measured with Metamorph software (Molecular Devices, Sunnyvale, CA) and tallied by hand from time-lapse microscopy images taken at 2 min intervals. The space around the cell, originating at the cell’s centroid, was divided into eight equal radial partitions with the anterior axis aligned with the longest initial cellular protrusion and fixed in this position. The orientation of protrusions was calculated based on the number of protrusions that were extended into each of the eight partitions over 12 hours. The polarization index, α, of cellular protrusions was calculated by equation 1,


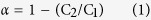


where C_2_ is the number of protrusions produced along the lateral axis of each cell over 12 hours, i.e. quadrants 3 & 7 in the orientation radial plot (refer to references^1^,^4^). C_1_ (quadrants 1 & 5) is the number of protrusions produced along the anteroposterior axis of the cell over 12 hours, with the anterior axis being set by the initial polarization of the cell at time zero. In effect, this parameter measures the re-orienting ability of the cellular protrusion away from their initial orientation over 12 hours. A value near 1 means the cell remains highly polarized along its initial polarization axis, whereas a value near zero indicates that the cell protrusions explored every angle equally. A One-Way Anova and Tukey post-test were used to calculate significance.

### Image pre-processing for reflection confocal collagen image

The non-uniform background, I_B_(x,y), in the raw image,(I_R_(x,y)) (Supplementary Fig. 2A), caused by interference patterns of the reflected incident laser light, was estimated by interpolating the intensity distribution as a function of radial distance from the center (I_B_(r)) of the image. The relation between intensity and radial distance from center of image was established from angular-averaged intensity at different radical distances. Raw reflection images were then normalized, I_N_(x, y), using the relationship in equation 2 below (Supplementary Fig. 2B), where < > is denoted for the mean value.


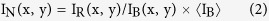


Further, we enhanced the fibrous structure in reflection confocal image by applying a fiber enhancement filtering (FEF) algorithm. The procedure for FEF in brief is stated below. At first, a mask filter (MSK_θ_) with size of 5 pixels by 5 pixels is created with a line across the center line at orientation θ. A 2-D anisotropic Gaussian filter (GF_θ_) was obtained from the normalized product of the line mask and was the same size (5 by 5) of the Gaussian filter. A 2-D anisotropic average filter (AF_θ_, 5 by 5in size) was also obtained from the normalized product of the line mask. The raw image was convolved with these two filters respectively to access the filtered image FEF_θ_, which is the result of subtracting the average filter convoluted image from the Gaussian filter convoluted image. FEF_θ_ at 15 different orientations distributed incrementally from 0 to 180 degree was used and the fiber enhanced filtering image (I_FEF_) is obtained from the maximum intensity projecting of FEF_θ_ (Supplementary Fig. 2C). The binary of FEF image (I_BW_) is obtained by setting a suitable threshold value (th) to the fiber enhance filtering image (I_FEF_) to highlight the collagen fibers (Supplementary Fig. 2D). The standard deviation for Gaussian filter was tested and found to be optimized at a value of 0.7. All image processing was conducted in MATLAB. All images were taken at the same magnification and size (1024 × 1024 pixels, 60x magnification) and were analyzed at the same size.

### Collagen pore size analysis

Relative differences in the mean pore size for the different concentration collagen matrices were estimated from the binarized reflection confocal images (refer to previous section for image pre-processing details, Supplementary Fig. 2) using a sequential morphological open operation based on techniques described by Gonzalez and Woods^48^ (Supplementary Fig. 3). First, the inverse or conjugate of the binary image was created, i.e. equation 3 below, to highlight the void regions between fibers (Supplementary Fig. 3A).


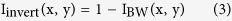


This conjugated image was then subjected to a sequential morphological opening operation of increasing width using a disk shape (Supplementary Fig. 3B–D). After morphological opening with a disk shape with radius of r, the remaining pore area, which is the summation of intensity in the image, I(r), represents the pore size with characteristic size larger than r (Supplementary Fig. 3E). Therefore, the areas in the image with pore size of radius equal to r are estimated by equation 4 below.


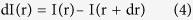


The frequency distribution at different pore sizes within an image was approximated by this distribution, equation 5 below.


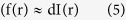


For each collagen concentration, the mean of the images’ pore size distributions were calculated and plotted (Fig. 2). Then these values were normalized within the set of varying collagen concentrations and plotted in correlation graphs (Figs 2 and 3).

### Collagen fiber alignment analysis

After pre-processing of the x, y reflection confocal images to enhance fiber detail (see “Image Pre-Processing…” section of Methods and Supplementary Fig. 2), the alignment of the fibers, i.e. the anisotropy of the white space in the resulting binary image (e.g. Supplementary Fig. 2D), was estimated using a discrete Fourier Transform (FT) method similar to that described by Sander and Barocas^49^. Broadly, this method measures the anisotropy (alignment) across the image of reflected light intensity peaks (fibers) after filtering and binarizing. Supplementary Fig. 4 shows example computer generated images and experimental pre and post-processed reflection images as well as their resulting polar FT plots and alignment values. FT allows the description of a spatially complex digital image to be represented in terms of the frequency of its components. The two-dimensional FT (Supplementary Fig. 4B,E) of a digital image of size M × N (Supplementary Fig. 4A,D) was calculated by equation 6 below,


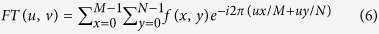


where x and y are the spatial coordinates of the digital image, u and v the frequency components along x and y in the Fourier domain, and 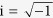. The magnitude of FFT (FT(u, v)) is calculated from equation 7,


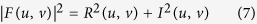


where R(u, v) and I(u, v) are the real and imaginary parts of FT(u, v). Then random transformation was applied to calculate the integrated intensity (F_I_ (θ)) over a line through the center at different orientations in the FT image. This Polar distribution of integrated FFT power was then transformed to Cartesian coordinates represented by equation 8 below (Supplementary Fig. 4C,F).


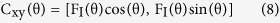


The two eigenvalue (λ_1_ and λ_2_, where λ_1_ > λ_2_) of the matrix [C_xy_^T^C_xy_] are used to calculate the alignment index, α, as α = 1 − λ_2_ /λ_1_. A value of α = 1 represents a completely aligned matrix of fibers, and α = 0 represents an isotropic matrix of fibers.

### Determination of elastic modulus

A strain-controlled rheometer with steel cone-and-plate geometry (25 mm in diameter; RFS3; TA Instruments) was used to measure viscoelastic properties of the varying concentrations of collagen matrices. First, collagen matrices were gelled between the cone and plate for 30 min at a temperature of 37 ^o^C in a humidified environment. A dynamic strain sweep was performed from 0.1% to 100% at a frequency of 1 rad/s to determine the region of linear elastic behavior, where the storage modulus G’ is independent of strain, i.e. below the critical strain where the network structure is disrupted. Three replicate experiments were performed for each collagen concentration, with new gels being formed for each replicate. Based on these results, a strain in the linear elastic regime for each collagen gel concentration was chosen (0.3% for 1–2 mg/ml, 0.8% for 2.5–6 mg/ml). Then new gels were made and a frequency sweep was performed at said strain from 0.1–100 rad/s. This was again repeated for three separate gels for each collagen concentration. The storage modulus was found to be independent of frequency in the range of ~6–100 rad/s for all concentrations, and this is the G’ that is reported.

### Statistics

The number of cells and biological repeats (n) for each experiment are indicated in the figure captions. Mean values ± s.e.m. and statistical analyses were calculated and plotted using Graphpad Prism (Graphpad Software, San Diego, CA). One-way analysis of variance and Tukey post-tests or pairwise two-tailed t-tests were conducted to determine significance, depending on the number of variables compared. Significance (p) was indicated within the figures using the following scale: *** for p < 0.001, ** for p < 0.01, and * for p < 0.05. Linear regressions were calculated and plotted using Graphpad Prism to visualize degree of correlation in correlation plots. Slope, square of the Pearson correlation coefficient (r^2^), and significance (p) of the regression’s deviation from zero slope and are shown on the plots.

## Additional Information

**How to cite this article**: Fraley, S. I. *et al*. Three-dimensional matrix fiber alignment modulates cell migration and MT1-MMP utility by spatially and temporally directing protrusions. *Sci. Rep*. **5**, 14580; doi: 10.1038/srep14580 (2015).

## Supplementary Material

Supplementary Information
