# Supplementary material for: Three-dimensional matrix fiber alignment modulates cell migration and MT1-MMP utility by spatially and temporally directing protrusions
Source: Sci Rep. 2015 Oct 1;5:14580. doi: 10.1038/srep14580 (PMC4589685; doi:10.1038/srep14580)
Supplement: Supplementary Information [file srep14580-s1.doc]

Three-dimensional matrix fiber alignment modulates cell migration and MT1-MMP utility by spatially and temporally directing protrusions

Stephanie I. Fraley*^a, b^**†, Pei-hsun Wu*^a, b^*, Lijuan He *^a, b^* , Yunfeng Feng *^b,c^*, Ranjini Krisnamurthy *^a^*, Gregory D. Longmore *^b^,* *^c^*& Denis Wirtz *^a, b^**

**Supplementary Figures:**

**Supplementary Figure 1. Cell motility controls and additional TGII data. A.** Graph showing the average cell speed of single MDA-MB-231 cells migrating within 3D collagen matrices of varying densities and matrix microstructures over a 24h observation time. Trend in migration speed mimics that shown by HT-1080 cells (Fig.1). **B.** Reflection confocal micrographs of untreated (bottom) and 10X TGII treated (top) 1 mg/ml collagen matrices. Scale bar is 10μm. **C.** Graph showing the average speed of single cells migrating on tissue culture treated or collagen-coated glass is unaffected by the presence of 10X TGII over a 16.5 h observation time. **D-F.** Correlation plots comparing invasion distance, 3D cell speed, and number of protrusions and including the outliers 4mg/ml and 6mg/ml. N=3 biological repeats for each experimental condition, at least 20 cells per repeat.

**
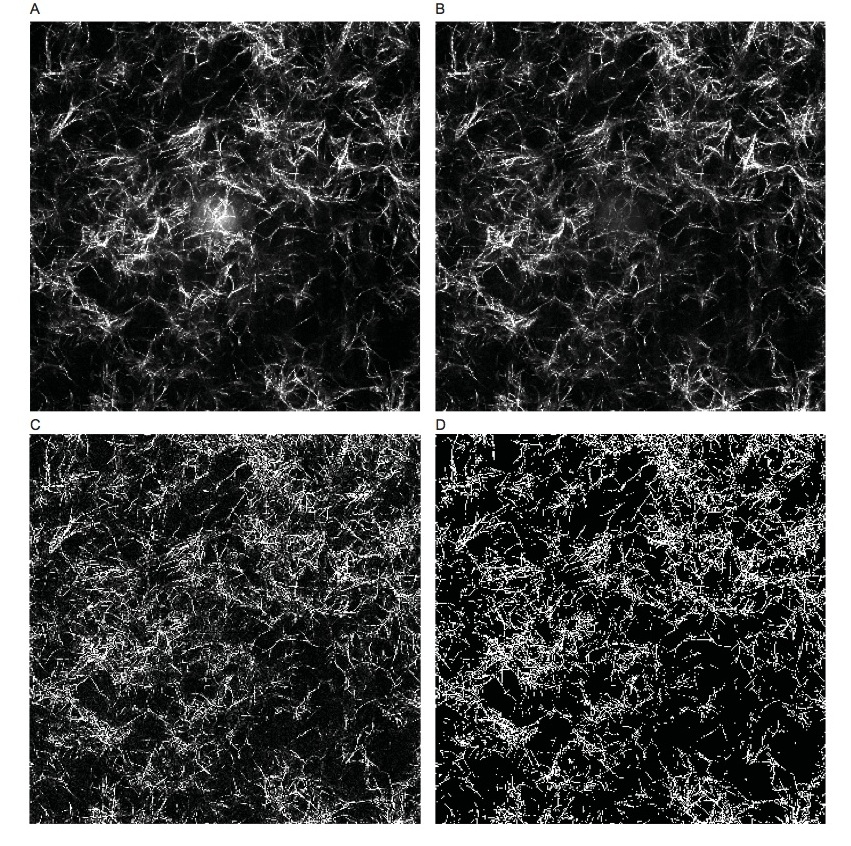
**

**Supplementary Figure 2. Normalization processing for reflection confocal images prior to alignment and interfiber spacing analysis. A.** Raw reflection confocal micrograph of collagen fibers, I_R_(x,y). **B.** Normalized reflection image, I_N_(x,y) after background has been subtracted. **C.** Normalized reflection image after fiber enhancement filtering has been applied, I_FEF_. **D.** Binarized version of C, I_BW_.

**
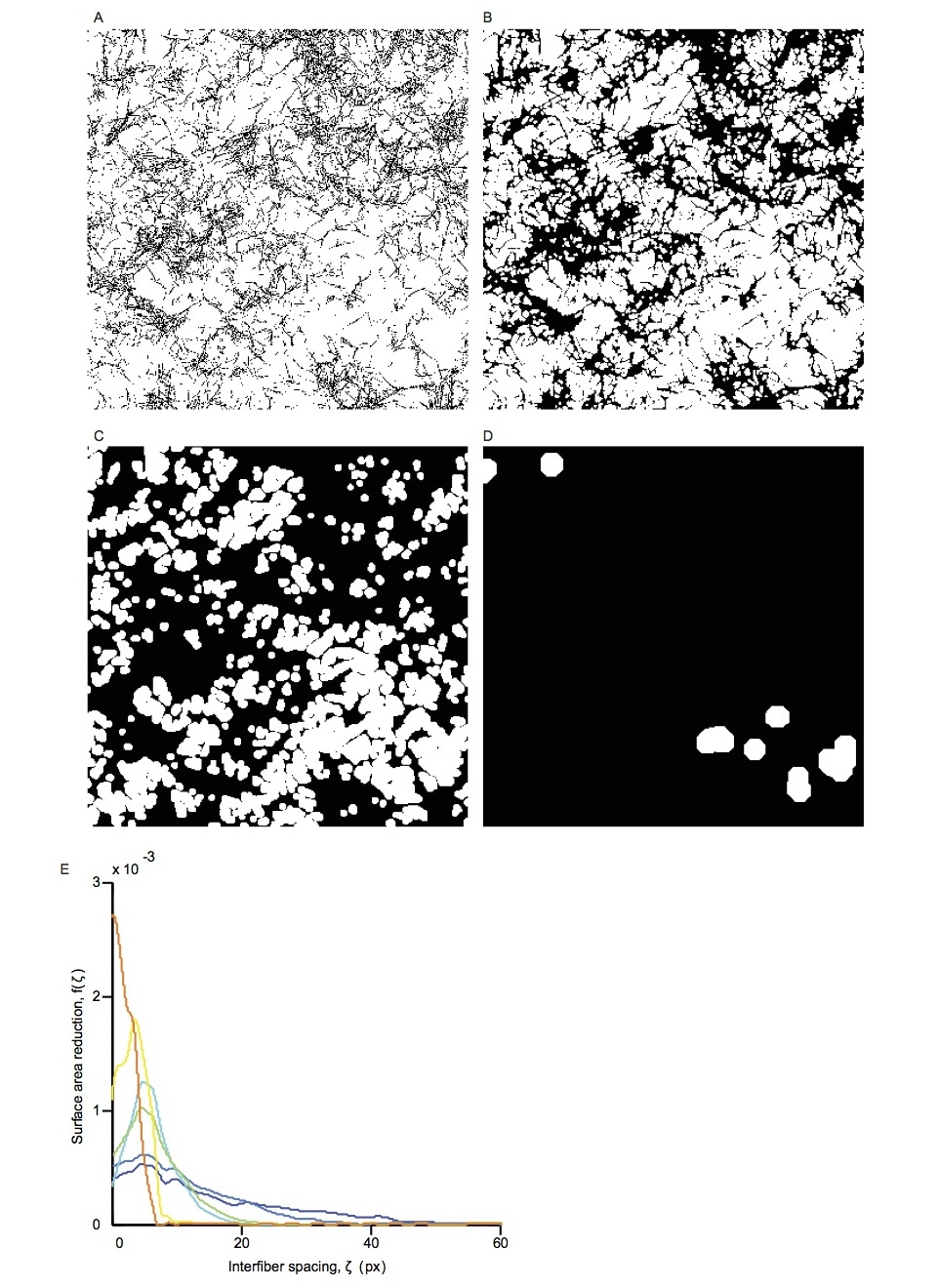
**

**Supplementary Figure 3. Pore size analysis. A.** Conjugate of binarized image for which intensity values have been inverted so that the pore is white and fibers are black, I_invert_(x,y). **B.** Image resulting from a morphological open processing applied to I_invert_(x,y) for w = 5 pixels; pores less than 5 pixels in radius have been removed. **C.** Image resulting from a morphological open processing applied to I_invert_(x,y) for w = 10 pixels; pores less than 10 pixels in radius have been removed. **D.** Image resulting from a morphological open processing applied to I_invert_(x,y) for w = 20 pixels; pores less than 20 pixels in radius have been removed. **E.** Graph showing the reduction in white surface area of the image as the morphological open process is applied iteratively with increasing w.


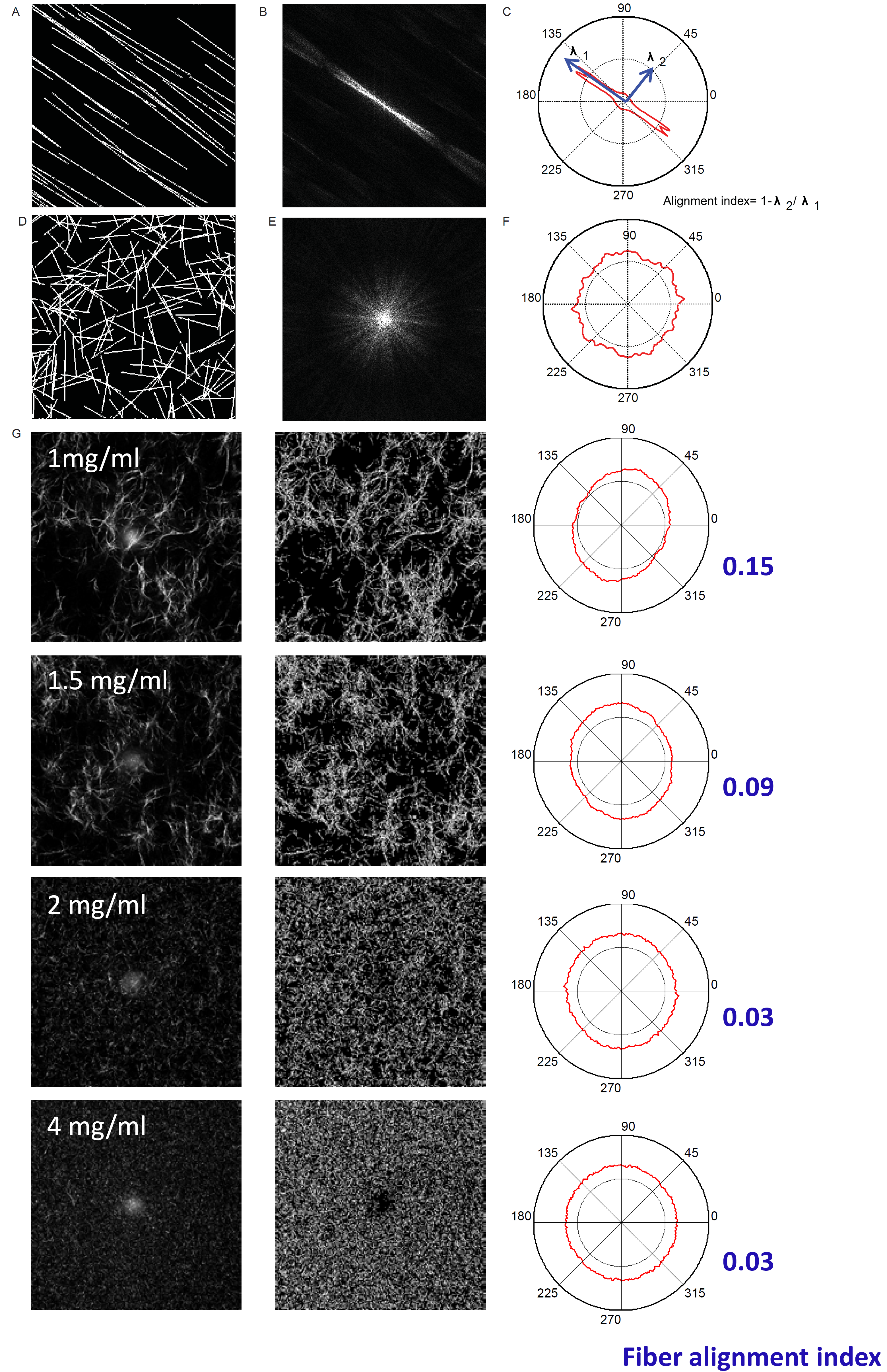


**Supplementary Figure 4. Fiber alignment analysis. A.** A computer generated image of highly aligned fibers. **B.** The FT of A. **C.** Polar plot of FT(Θ) of A showing the distribution of intensity frequency, which peaks at 137^o^. Eigenvalues used to calculate alignment index are indicated by blue arrows. Here, the alignment index is close to 1.  **D.** A computer generated image of isotropically arranged fibers. **E.** The FT of D. **F.** Polar plot of FT(Θ) of D showing the distribution of intensity frequency, which has no major peaks. Here the alignment index is close to 0. **G.** Example experimental reflection confocal images of collagen fibers: raw (left column), after background subtraction, fiber enhancement, and conversion to binary (middle column), and their associated polar plots of FT(Θ) with alignment index value printed in blue (right column).
